# Hyperbaric Oxygen Therapy Improves the Osteogenic and Vasculogenic Properties of Mesenchymal Stem Cells in the Presence of Inflammation In Vitro

**DOI:** 10.3390/ijms21041452

**Published:** 2020-02-20

**Authors:** Chiara Gardin, Gerardo Bosco, Letizia Ferroni, Silvia Quartesan, Alex Rizzato, Marco Tatullo, Barbara Zavan

**Affiliations:** 1 Maria Cecilia Hospital, GVM Care & Research, 48033 Cotignola (RA), Italy; chiara.gardin@unife.it (C.G.); letizia.ferroni@unife.it (L.F.); 2 Department of Medical Sciences, University of Ferrara, 44121 Ferrara, Italy; 3 Department of Biomedical Sciences, University of Padova, 35128 Padova, Italy; gerardo.bosco@unipd.it (G.B.); silvia.quartesan@gmail.com (S.Q.); alex.rizzato@unipd.it (A.R.); 4 Department of Basic Medical Sciences, Neurosciences and Sense Organs, University of Bari “Aldo Moro”, 70121 Bari, Italy

**Keywords:** adipose-derived stem cells, hyperbaric oxygen therapy, inflammation, osteogenic differentiation, vasculogenic differentiation, bone regeneration

## Abstract

Hyperbaric oxygen (HBO) therapy has been reported to be beneficial for treating many conditions of inflammation-associated bone loss. The aim of this work was to in vitro investigate the effect of HBO in the course of osteogenesis of human Mesenchymal Stem Cells (MSCs) grown in a simulated pro-inflammatory environment. Cells were cultured with osteogenic differentiation factors in the presence or not of the pro-inflammatory cytokine Tumor Necrosis Factor-*α* (TNF-*α*), and simultaneously exposed daily for 60 min, and up to 21 days, at 2,4 atmosphere absolute (ATA) and 100% O_2_. To elucidate osteogenic differentiation-dependent effects, cells were additionally pre-committed prior to treatments. Cell metabolic activity was evaluated by means of the MTT assay and DNA content quantification, whereas osteogenic and vasculogenic differentiation was assessed by quantification of extracellular calcium deposition and gene expression analysis. Metabolic activity and osteogenic properties of cells did not differ between HBO, high pressure (HB) alone, or high oxygen (HO) alone and control if cells were pre-differentiated to the osteogenic lineage. In contrast, when treatments started contextually to the osteogenic differentiation of the cells, a significant reduction in cell metabolic activity first, and in mineral deposition at later time points, were observed in the HBO-treated group. Interestingly, TNF-*α* supplementation determined a significant improvement in the osteogenic capacity of cells subjected to HBO, which was not observed in TNF-*α*-treated cells exposed to HB or HO alone. This study suggests that exposure of osteogenic-differentiating MSCs to HBO under in vitro simulated inflammatory conditions enhances differentiation towards the osteogenic phenotype, providing evidence of the potential application of HBO in all those processes requiring bone regeneration.

## 1. Introduction

Bone loss and subsequent repair are biological processes related to many pathological conditions affecting bones, including fractures, osteoporosis, osteoarthritis, osteomyelitis, osteonecrosis, and tumors [1]. Current treatments are typically invasive and, depending on the bone-affecting disease, they involve grafting, surgery, debridement, and/or pharmacotherapy [2]. Recently, hyperbaric oxygen (HBO) has been proposed as an adjunctive therapy in the management of several conditions requiring bone healing [3,4,5,6]. The HBO therapy is the noninvasive administration of 100% O_2_ at pressures greater than one atmosphere absolute (ATA). Treatments usually involve pressurization between 1.5 and 3.0 ATA inside airtight chambers for periods between 60 and 120 min once or twice daily [7]. In this way, it is possible to deliver a greatly increased supply of oxygen to tissues, which is advantageous for fibroblasts proliferation, collagen fiber deposition, and formation of new blood vessels, thus accelerating tissue repair and healing, and easing pain [8]. In addition, high-pressure oxygen can enhance the viability and capacity of phagocytic cells, which are beneficial to the absorption and removal of necrotic tissues, and reduction of local inflammation [9].

Inflammation also represents a crucial event in the process of bone repair, being the initial inflammatory response necessary for the regeneration to progress [10]. In fact, inflammatory stimuli determine the recruitment of Mesenchymal Stem Cells (MSCs), and direct their migration, proliferation and differentiation into mature osteoblasts [11]. At the same time, MSCs modulate inflammatory cells to promote resolution of pro-inflammatory activities, and reconstitution of normal tissue [12]. Tumor Necrosis factor-*α* (TNF-*α*) is among the inflammatory mediators more significantly elevated after bone injury [13]. Nevertheless, prolonged or chronic expression of these inflammatory molecules has negative effects on bone, leading to increased bone resorption and suppressed bone formation [13]. A clear example of bone loss determined by a chronic inflammatory state is represented by periodontitis. In this multifactorial disease, levels of different inflammatory mediators, such as interleukin-1 (IL-1), IL-6, prostaglandins, C-reactive protein, as well as TNF-*α*, are found elevated in patients’ serum [14]. In the long term, this prolonged inflammatory condition causes the tooth’s supporting tissues, in particular the alveolar bone, to be destroyed, leading to the final loss of the tooth. Several studies have evidenced a positive correlation between periodontal disease and cardiovascular disease (CVD), including coronary heart disease, stroke, and endothelial dysfunction [15,16]. In very recent works, numerous molecules have been identified in serum and saliva of patients suffering from both periodontitis and CVD, in particular high levels of endothelin-1 and asymmetric dimethylarginine, and low levels of vitamin C and antioxidants, which can be used as novel biomarkers for predicting the development of these different but related diseases [17,18,19].

In the light of these observations, the rationale of the current study was to determine whether HBO or its individual constituents, elevated pressure or elevated oxygen, could influence different cellular functions, and if these change depending on the inflammatory microenvironment in which the cells reside. In detail, the aim of the present work was to analyze the direct effects of HBO on the metabolic activity and on the osteogenic and vasculogenic differentiation properties of human Adipose-Derived Stem Cells (hADSCs) grown in a simulated pro-inflammatory environment. MSCs isolated from human adipose tissue samples share many properties with those harvested from bone marrow, especially the same differentiation potential and a similar surface markers expression [20]. In addition, hADSCs represent a source of several cytokines and soluble factors with angiogenic, antiapoptotic, and antioxidative properties, and also act as modulators of the immune system, by inhibiting the secretion of inflammatory cytokines while stimulating the production of those with anti-inflammatory activity [21]. As of these properties and because these cells can be easily harvested in great amounts with minimal donor-site morbidity and without ethical concerns, hADSCs have proved to be particularly promising for bone repair and regeneration therapies [22].

In this study, the inflammatory environment was recreated in vitro by exposing hADSCs to the pro-inflammatory cytokine TNF-α for all the duration of the osteogenic differentiation. Contextually, cells were treated daily for 60 min, and up to 21 days, at 2,4 ATA and 100% O_2_. The influence of the individual stimuli, high pressure or high oxygen, on stem cells functions was also explored. In order to evaluate the effect of HBO during the osteogenic differentiation process, hADSCs were additionally pre-exposed to osteogenic factors for 10 days before starting treatments. Then, cell metabolic activity was evaluated by means of the MTT assay and measurement of DNA content, whereas osteogenic differentiation was assessed by quantification of extracellular calcium deposition after Alizarin Red S (ARS) staining and gene expression analysis of selected osteogenic markers. Real-time PCR was employed for additionally evaluating the expression of some vasculogenic markers. The results of our work suggest that osteogenic-differentiating cells respond differently to pressure and oxygen signals, in particular under an inflammatory environment. Notably, the combination of high pressure and high oxygen seems to favor hADSCs osteogenesis during the prolonged inflammatory state, providing evidence of the potential therapeutic use of HBO in all those physiological processes that require bone regeneration.

## 2. Results

### 2.1. Effect of HBO on hADSCs Metabolic Activity

**Figure 1:**
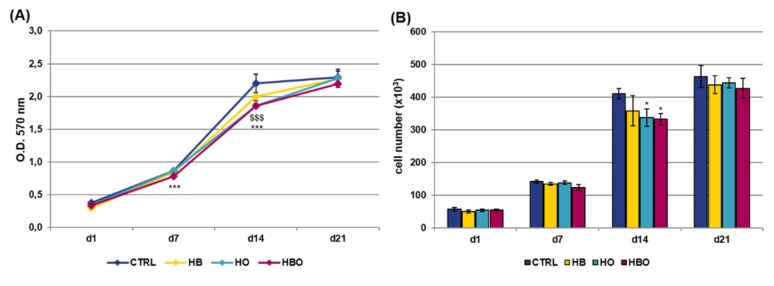
Metabolic activity and proliferation of human Adipose-Derived Stem Cells (hADSCs) after hyperbaric oxygen (HBO) treatment. (**A**) MTT assay and (**B**) quantification of DNA content in hADSCs 1 day (d1), 7 days (d7), 14 days (d14), and 21 days (d21) after osteogenic differentiation in Osteogenic Differentiation Medium (ODM) and hyperbaric oxygen (HBO), hyperbarism (HB), or hyperoxia (HO) treatment. The control condition (CTRL) is represented by cells grown under normoxia and normobarism. Data are shown as mean ± standard deviation (SD). * *p* < 0.05, *** and $$$ *p* < 0.001 indicate statistically significant difference between the HBO- or HO-treated cells and the control group, respectively.

The MTT assay and quantification of DNA content were performed at selected time points over a period of 21 days to compare the metabolic activity of hADSCs exposed to different pressure and oxygen signals. As shown in Figure 1A, untreated cells showed an increase in proliferation up to 14 days of culture; then, these stopped to proliferate and reached the plateau phase at day 21. Compared with the control group, daily HBO treatment determined a significant (*p* < 0.001) decrease in the rate of hADSCs proliferation both at day 7 and day 14. In a similar way to HBO, cell metabolic activity was significantly (*p* < 0.001) reduced in the HO-treated cultures at day 14. On the contrary, treatment with high pressure alone did not induce any significant change in cell metabolic activity compared with the control. In any case, at day 21, all the cell cultures reached confluence and no difference in cell proliferation was noted between the treated and untreated groups. These trends were confirmed by quantification of DNA content at the different culture time (Figure 1B).

**Figure 2:**
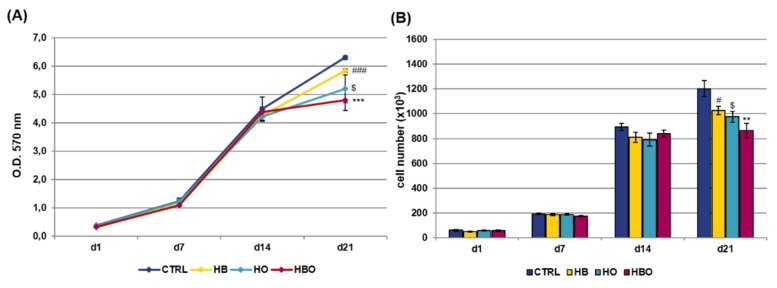
Metabolic activity and proliferation of hADSCs after HBO treatment in the presence of inflammation. (**A**) MTT assay and (**B**) quantification of DNA content in hADSCs 1, 7, 14, and 21 days after osteogenic differentiation in the presence of 10 ng/mL Tumor Necrosis factor-*α* (TNF-*α*) and HBO, HB, or HO treatment. Data are shown as mean ± SD. In both graphs, # and $ *p* < 0.05 indicate statistically significant difference between the HB- or HO-treated cells and control. ** *p* < 0.01 and *** *p* < 0.001 indicate statistically significant difference between the HBO-treated cells and the control group; ### p < 0.001 indicates statistically significant difference between the HB-treated cells and the control group.

When the pro-inflammatory cytokine TNF-*α* was added to the Osteogenic Differentiation Medium (ODM) contextually to the beginning of treatments, the results of the MTT assay (Figure 2A) and DNA content quantification (Figure 2B) revealed that hADSCs exposed to HBO, HB or HO had comparable growth rates with respect to the control condition up to day 14. However, at day 21, a significant reduction in cell metabolic activity was observed in all the three treated groups.

### 2.2. Effect of HBO on Extracellular Matrix Mineralization

**Figure 3:**
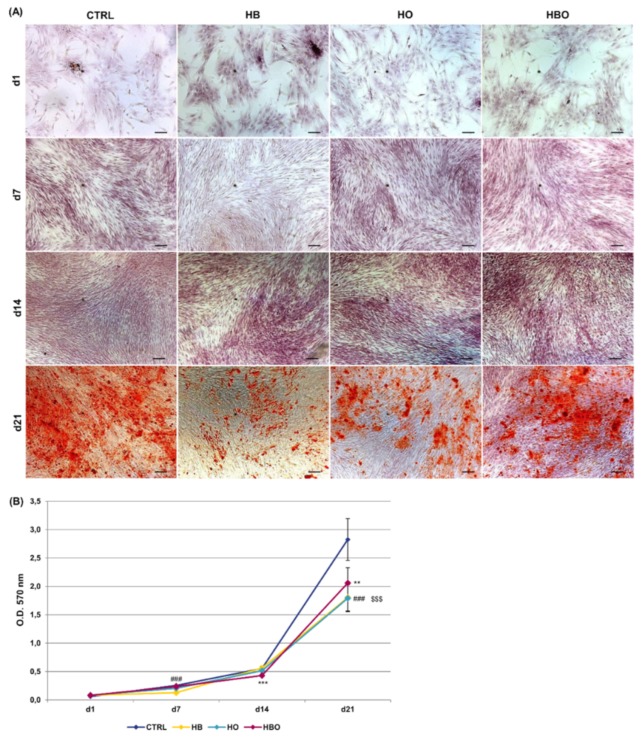
Matrix mineralization in hADSCs after HBO treatment. (**A**) Alizarin Red S (ARS) staining and (**B**) quantification of calcium deposits in hADSCs after d1, d7, d14, and d21 of culture in ODM and HBO, HB, or HO treatment. Scale bars 100 µm. Data are shown as mean ± SD. ** *p* < 0.01 and *** *p* < 0.001 indicate statistically significant difference between the HBO-treated cells and the control group, respectively. ### and $$$ *p* < 0.001 indicate statistically significant difference between HB- and HO-treated cells and control, respectively.

Staining of calcium deposits in the extracellular matrix followed by quantification of extracted ARS is reported in Figure 3. ARS accumulation in cells was detectable after 21 days of osteogenic differentiation both in the control and in the HBO-, HB-, or HO-treated groups (Figure 3A). Nevertheless, quantification of ARS stain by Cetylpyridinium Chloride (CPC) extraction revealed that levels of calcium deposition were significantly lower for all the treated cultures compared with the untreated control group (Figure 3B). Notably, a significant (*p* < 0.001) reduction in ARS quantification was noticeable already at day 7 for the HB-treated cultures, and at day 14 for the HBO group.

**Figure 4:**
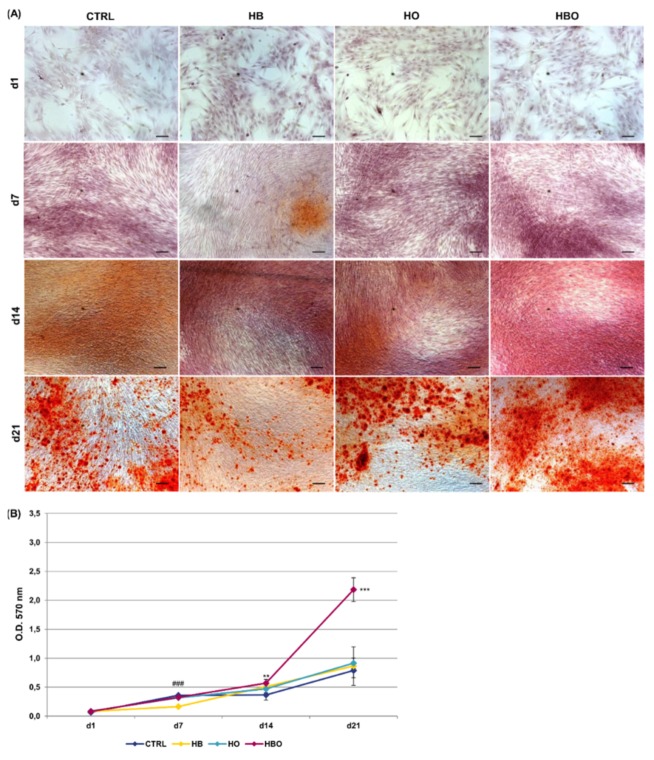
Matrix mineralization in hADSCs after HBO treatment in the presence of inflammation. (**A**) ARS staining and (**B**) quantification of calcium deposits in hADSCs after d1, d7, d14, and d21 of culture in ODM supplemented with 10 ng/mL TNF-*α* and HBO, HB, or HO treatment. Scale bars 100 µm. Data are shown as mean ± SD. ** *p* < 0.01 and *** *p* < 0.001 indicate statistically significant difference between the HBO-treated cells and the control group, respectively. ### *p* < 0.001 indicate statistically significant difference between the HB-treated cells and control.

ARS accumulation was visible at day 21 of osteogenic differentiation for all the tested conditions, even when the cells were cultured in the presence of the pro-inflammatory cytokine TNF-*α* (Figure 4A). Nevertheless, under in vitro simulated inflammatory conditions level of calcium deposition was significantly (*p* < 0.001) higher in cells subjected to the HBO treatment compared to control (Figure 4B). On the contrary, no differences were detected in the amount of mineral nodules formation between treatments with elevated pressure or elevated oxygen alone and the control group at day 21. Compared to control, extracellular calcium deposition in the HBO-treated group started to increase significantly (*p* < 0.01) at 14 days of culture.

### 2.3. Effect of HBO on Pre-Committed hADSCs

**Figure 5:**
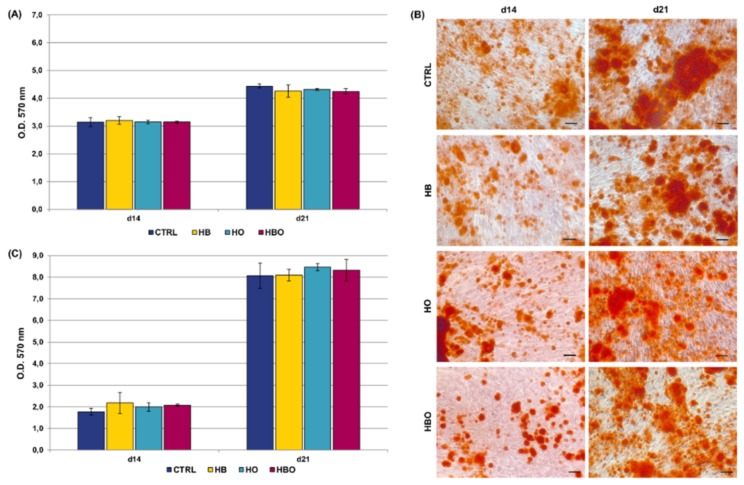
Metabolic activity and matrix mineralization of pre-committed hADSCs. (**A**) MTT assay of hADSCs 14 and 21 days after osteogenic differentiation in ODM and HBO, HB, or HO treatment. Data are shown as mean ± SD (*n* = 3). (**B**) ARS staining and (**C**), quantification of calcium deposits in hADSCs after d14 and d21 of culture in ODM and HBO, HB, or HO treatment. Scale bars 200 µm. Data are shown as mean ± SD (*n* = 3).

Measurement of metabolic activity of hADSCs pre-differentiated for 10 days in ODM before starting treatments revealed that HBO did not affect cell proliferation when compared to the untreated condition at 14 and 21 days of culture; this was also true for cells subjected to the HB or HO treatments alone (Figure 5A). Quantification of mineral calcium deposits by hADSCs pre-exposed to osteogenic stimuli showed a considerable increment with culture time in all groups, without any difference between treatments and control (Figure 5B,C).

### 2.4. Effect of HBO on Expression of Osteogenic and Vasculogenic Markers by hADSCs

**Figure 6:**
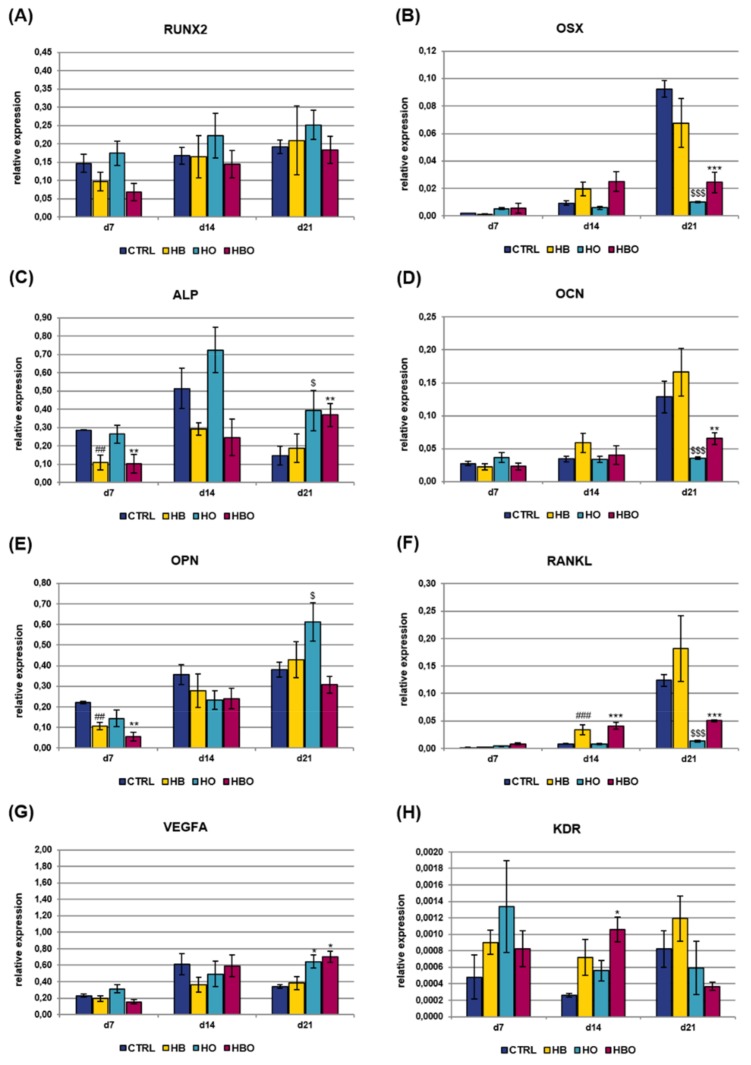
Gene expression profile of osteogenic and vasculogenic markers in hADSCs after HBO treatment. Expression of the osteoblast lineage genes (**A**) RUNX2; (**B**) OSX; (**C**) ALP; (**D**) OCN; (**E**) OPN; (**F**) RANKL; (**G**) VEGFA; and (**H**) KDR measured by real-time PCR. Data are expressed as mean ± SD of the target gene versus reference gene (transferrin receptor, TFRC) ratio and represented by 2deltaCt. *, $ *p* < 0.05, ##, ** *p* < 0.01, and ###, $$$, ***p < 0.001 mark significant changes in gene expression level compared to the control within the same measurement day.

Real-time PCR was used to monitor the expression of key osteoblast regulators and bone matrix proteins in hADSCs subjected to HBO-, HB-, or HO-treatment from the beginning of osteogenic differentiation. Analyzed osteogenic markers included runt related transcription factor 2 (RUNX2), osterix (OSX), alkaline phosphatase, liver/bone/kidney (ALP), osteocalcin (OCN), osteopontin (OPN), and tumor necrosis factor (ligand) superfamily, member 11 (RANKL); gene expression of vascular endothelial growth factor A (VEGFA) and kinase insert domain receptor (KDR) was additionally evaluated (Figure 6). TFRC was chosen as reference gene for real-time PCR data normalization since its expression resulted stable during MSCs osteogenic differentiation, as also established in our previous works [23,24]. The levels of gene expression for the key transcription factor RUNX2 slightly increased with culture time both in the treated and control groups, without significant differences between the various conditions (Figure 6A). Expression of the other transcriptional regulator, OSX, was found to be significantly (*p* < 0.001) reduced in the HBO-treated cultures as well as in the HO group at day 21 (Figure 6B). The expression of ALP, an early marker for osteogenic differentiation, peaked after 14 days of culture in ODM in the untreated control cells, then it decreased at day 21 (Figure 6C). In contrast, when hADSCs underwent the HBO treatment, ALP expression levels were significantly (*p* < 0.01) lower than control on day 7, then they gradually increased over time, reaching the maximum after 21 days of culture. ALP gene expression for the HB- and HO-treated groups trended the same as described for the control cultures. The expression of the mineralization-specific genes OCN and OPN was further evaluated. HBO, elevated pressure or elevated oxygen alone had no effect on OCN expression compared to control until day 14; nevertheless, at day 21, OCN expression significantly decreased in the HBO- and in the HO-treated groups (*p* < 0.01 and *p* < 0.001, respectively) when compared to control (Figure 6D). The expression of the specific matrix protein OPN showed a gradual increase over time in all cell cultures, but a significant (*p* < 0.01) down-regulation following treatment with HBO and HB was observed at day 7 (Figure 6E). RANKL expression levels were not significantly different at day 7 of differentiation for all the tested conditions, but noticeable differences were evidenced in the course of culture time (Figure 6F). In particular, at day 14, HBO and HB significantly (*p* < 0.001) increased RANKL expression when compared to control cultures, whereas elevated oxygen alone had no effect. Nevertheless, a significant (*p* < 0.001) down-regulation of RANKL expression was observed for HBO and HO after 21 days of osteogenic differentiation. VEGFA expression was similar in the control and treated groups during the first 14 days of culture; nevertheless, a significant (*p* < 0.05) up-regulation was monitored in cells treated with HBO or HO at day 21 (Figure 6G). The expression of the VEGFA receptor, KDR, significantly (*p* < 0.05) increased in HBO-treated cells at day 14; whereas it was comparable and without any significant difference between the other tested conditions at any time during culture (Figure 6H).

**Figure 7:**
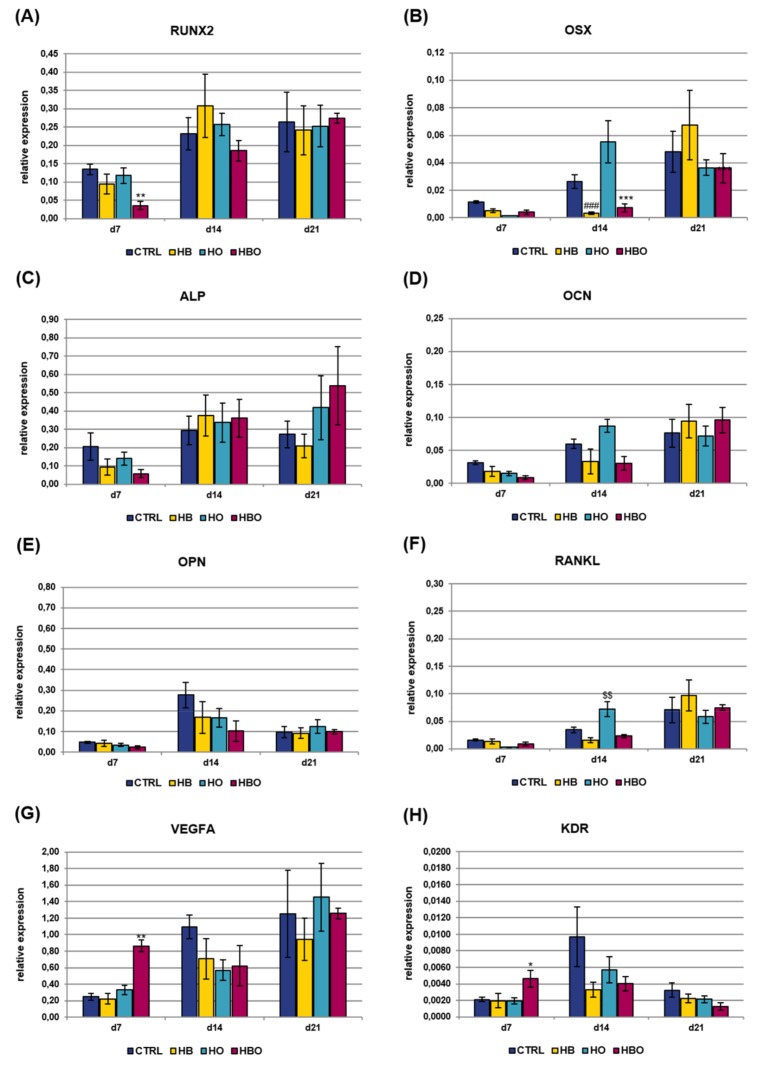
Gene expression profile of osteogenic and vasculogenic markers in hADSCs after HBO treatment in the presence of inflammation. Expression of (**A**) RUNX2; (**B**) OSX; (**C**) ALP; (**D**) OCN; (**E**) OPN; (**F**) RANKL; (**G**) VEGFA; and (**H**) KDR measured by real-time PCR. Data are expressed as mean ± SD of the target gene versus reference gene (TFRC) ratio and represented by 2deltaCt. * *p* < 0.05, **, $$ *p* < 0.01, and ***, ### *p* < 0.001 mark significant changes in gene expression level compared to the control within the same measurement day.

Gene expression of osteogenic and vasculogenic markers was additionally quantified in cell cultures exposed to the pro-inflammatory cytokine TNF-*α* for all the duration of differentiation (Figure 7). The expression of RUNX2 showed a gradual increase with culture time both in the control and treated groups, with a significant (*p* < 0.01) difference between HBO and control observed at day 7 (Figure 7A). Also OSX expression levels increased with culture time in all the tested conditions, although a significant (*p* < 0.001) reduction was measured in the HBO- and HB-treated groups respect to control at day 14 (Figure 7B). The expression of ALP and OCN increased in the HBO-treated group as well as in the control over time, similarly to what observed in cells exposed to elevated pressure or elevated oxygen alone (Figure 7C and 7D). The expression profile of OPN was comparable and without any significant difference between the tested conditions at any time during culture (Figure 7E). RANKL expression trended the same in the HBO- and HB-treated groups when compared to control at each culture time; in contrast, a significant (*p* < 0.01) up-regulation in its transcriptional levels was monitored in cells subjected to elevated oxygen alone at day 14 (Figure 7F). For both VEGFA (Figure 7G) and KDR (Figure 7H), a significant (*p* < 0.01 and *p* < 0.05, respectively) up-regulation of their expression levels was noted after 7 days of culture in HBO-treated cells.

## 3. Discussion

A considerable number of studies have shown enhanced osteogenic activity on human osteoblasts and bone marrow MSCs (BM-MSCs) as a result of HBO treatment [25,26,27,28]. In particular, it has been reported that HBO promotes osteogenesis by stimulating osteoblast activity, neo-angiogenesis, and increasing the accumulation of minerals needed for osteogenesis, such as calcium, magnesium, and phosphorus [25,26,29]. To the best of our knowledge, the direct effects of the HBO therapy on metabolic activity and osteogenic differentiation of hADSCs grown in a simulated pro-inflammatory environment have not been investigated previously. In this study, hADSCs isolated from human adipose tissue samples, then cultured and differentiated following the same protocol published in a recent work from our laboratory, were used [23]. In that work, flow cytometric immunophenotypic characterization showed that >99% of cells were positive for the MSCs-specific markers CD44, CD73, CD90, and CD105, and simultaneously negative for the hematopoietic markers CD14, CD34, and CD45. Once isolated, hADSCs were induced to osteogenic differentiation and contextually exposed to HBO, HB or HO daily for 60 min, with the aim to replicate the duration of treatment received by hyperbaric therapy patients [3]. Each treatment was carried out for 21 days, which is generally considered an appropriate time period for in vitro evaluating the osteogenic differentiation properties of hADSCs [30].

In human tissues, MSCs reside in an hypoxic microenvironment, containing oxygen tensions ranging between 2% and 9%, which are considerably different from the inspired ambient oxygen levels of 21% (160 mm Hg) [31]. This is because there is a distribution of oxygen tensions according to a gradient [32]. As the inhaled air passes from the lungs to the circulation, the oxygen partial pressure (pO_2_) progressively drops to about 14–65 mm Hg, depending on the target tissue, corresponding to the mentioned levels of 2–9% O_2_ [31,33,34]. This also occurs at the cellular level [32]. It has been widely demonstrated that such hypoxic conditions contribute to maintaining MSCs in an undifferentiated and multipotent status [34,35,36]. Nonetheless, the osteogenic differentiation potential is found diminished or even impaired when these cells are grown in hypoxic conditions [37,38,39]. During HBO treatment (100% O_2_), the PaO_2_ in tissues rises to around 1500 mm Hg, which corresponds to pO_2_ of 500 mm Hg in soft tissue and 200 mm Hg in bone [4]. It is well accepted that such exposure to high oxygen concentrations increases the formation of reactive oxygen species (ROS), which in turn results in consumption of antioxidants and reduction of antioxidant enzyme activity [40]. Furthermore, ROS can react with macromolecular components, inducing cells necrosis or apoptosis [41]. However, the effects generated by ROS can be either positive or negative, depending on their concentration and intracellular localization [42]. Several studies have demonstrated that, since exposure to hyperoxia in clinical HBO therapy is rather brief, cellular antioxidant defenses are adequate and biochemical stress related to increased ROS is reversible [43,44]. More interestingly, it has been reported that the initial oxidative stress induced by HBO therapy acts as a trigger mechanism stimulating antioxidative and anti-inflammatory responses in cells [45].

In this work, measurements of the cell metabolic activity by means of the MTT assay revealed that daily HBO treatment significantly decreases the rate of hADSCs proliferation both at 7 and 14 days respect to untreated control cells. Further quantification of DNA content confirmed a reduced proliferation for the HBO-treated cells at 14 days. This difference disappeared by day 21, when all the cell cultures reached confluence. MTT assay measurements are indicative of cell metabolism, since the method is based on the reduction of a tetrazolium compound by mitochondrial dehydrogenase enzymes present in metabolically active cells [46]. The reduction we observed at both 7 and 14 days in HBO-treated hADSCs thus provides an indication of a decreased metabolic activity by cells, which in turn could depend both on the reduction in cell number or on the reduction in the mitochondrial activity per cell [47]. Nevertheless, this effect was neutralized at day 21, when hADSCs treated with HBO showed metabolic activity comparable to control cells. Looking at the literature, variable responses of HBO on cell proliferation have been reported [25,26]. In agreement with our results, the study of Wong on primary human osteoblasts demonstrated that daily HBO treatments caused a 24% decrease in cell growth after 9 days in culture, through induction of apoptosis and cell cycle arrest [47]. In another study, Lin and colleagues evaluated the role of HBO on the proliferation rate of BM-MSCs, induced or not to the osteogenic lineage for up to 14 days [27]. Interestingly, the authors found that HBO increased proliferation in osteogenically differentiated cells, whereas a decrease in cell proliferation following HBO treatment was noted in uncommitted BM-MSCs. However, none of the studies published so far evaluated the effect of HBO on hADSCs proliferation for 21 consecutive days. High pressure alone, but not high oxygen alone, did not have any effect on cell metabolic activity, similarly to untreated control cells.

The metabolic activity and proliferation of cells exposed to different pressure and oxygen signals was additionally evaluated under in vitro simulated inflammatory conditions. The environment in which MSCs reside, indeed, has been shown to influence the behavior and differentiation of these cells, in addition to their regenerative capabilities [12]. In this regard, in all those situations where new bone needs to be formed or repaired, for example as a result of damage, fracture, or around prosthetic implants, the milieu contains many inflammatory cytokines, due to the concomitant damage and host response to such damage [1]. In this work, inflammatory conditions were simulated in vitro by supplementing the ODM with the pro-inflammatory cytokine TNF-*α* at a concentration of 10 ng/mL. By comparing the growth rate of cells cultured in the presence or not of TNF-*α* within each condition, a substantial increase in cell proliferation was recorded in the TNF-*α*-treated groups at days 14 and 21, suggesting some mitogenic effects induced by the cytokine supplementation. The effect of TNF-*α* on hMSCs proliferation is well documented in the literature, and it seems to be strongly dependent by its concentration [48,49]. Bastidas-Coral and colleagues, for example, reported that TNF-*α* at a relatively low concentration of 10 ng/mL increases hADSCs proliferation after 7 days of culture, suggesting an important role of the cytokine during bone tissue repair [48]. In a previous study, Egea and coworkers demonstrated that 50 ng/mL TNF-*α* for 7 days does not affect MSCs proliferation, whereas lower dosages of the cytokine significantly stimulate MSCs proliferation, with a 2-fold increment at a concentration of 5 ng/mL [49].

When hADSCs are treated with TNF-*α* and contextually exposed to HBO, HB or HO, comparable growth rates with respect to the control condition were noted up to day 14. However, at day 21, a significant reduction in cell metabolic activity, as revealed by MTT assay, and in cell proliferation, measured in terms of DNA content, were observed in all the three treated groups. So far, no studies evaluating the combinatorial effect of HBO and TNF-*α* on the metabolic activity of hADSCs have been published. The only work that examined the simultaneous effect of these two parameters on cell proliferation employed bone marrow-derived Endothelial Progenitor Cells, that were pre-exposed to the cytokine incubation before applying HBO [50]. Overall, considering the results of MTT assay and DNA content, the observed reduced cell proliferation following HBO treatment could be explained by induction of cell death or cell cycle arrest through the generation of ROS [51]. As interestingly reviewed by Chandel and Budinger, these increased ROS result in activation of Bcl-2 pro-apoptotic proteins, which eventually lead to mitochondrial membrane permeabilization and cell death [52]. Nevertheless, further investigations would be necessary for elucidating the exact mechanisms through which HBO influences hADSCs growth.

The reduced proliferation rate at later time points can also be regarded as a sign of progression in differentiation [53]. We therefore monitored the effects of the HBO treatment during hADSCs osteogenesis by measuring calcium deposition in the extracellular matrix, which is considered as one of the main parameters to assess osteogenic differentiation in vitro [54]. Matrix mineralization begins a few days after the initial accumulation of osteoblast proteins, such as collagen and ALP, along with calcium and phosphate deposition up to the late stage of mineralization [55]. The ARS staining performed on treated and untreated cells confirmed the progression of osteogenic differentiation from days 1 to 21. Nevertheless, quantification of the staining revealed a significant reduction in mineral deposition in the HBO-, HB-, and HO-treated cells compared to the untreated control at day 21, indicating that pressure and oxygen signals somewhat interfere with the progression of hADSCs osteogenic differentiation. Our results are in contrast with those of Lin and colleagues, which reported that HBO significantly increases calcium levels after 14 and 21 days of BM-MSCs osteogenic induction compared to the control group [27,28]. The discrepancies among the present results and those reported by Lin and coworkers may arise from the use of different cells (ADSCs versus BM-MSCs), different treatment durations (60 min once daily versus 90 min every 36 h), and different calcium detection methods (calcium mineral deposits versus free calcium ions). The trend was reversed in the presence of inflammation, where cytokine supplementation associated to osteogenic factors determined a reduction in mineral matrix deposition in untreated cells respect to control cells not exposed to TNF-*α* after 21 days of culture. In agreement with our results, the study of Bastidas-Coral and colleagues reported that exposure of hADSCs to 10 ng/mL TNF-*α* decreases matrix mineralization compared to untreated cells after 14 days of culture [48]. Despite several other works investigating the influence of TNF-*α* on osteogenic differentiation of MSCs, its role still remains controversial. Indeed, both stimulatory and inhibitory effects on MSCs osteoblastogenesis have been described for the pro-inflammatory cytokine [54,56,57,58,59]. Most of these studies, however, agree on the fact that TNF-*α* influences the MSCs osteogenic differentiation in relation to cytokine concentration, timing and duration of administration, as well as to the cell types and species used [60,61,62,63]. Once again, no studies so far analyzed the simultaneous effect of HBO and TNF-*α* on hADSCs osteogenesis.

The most relevant finding, however, is that, in the presence of prolonged inflammation, the combination of elevated pressure and elevated oxygen was able to increase matrix mineralization at levels higher than those seen in normobaric and normoxic controls. Notably, the increment in extracellular calcium deposition was already evident after 14 days of culture. In contrast, daily treatments with HB or HO alone did not have an effect on bone nodule formation, therefore being unable to overcome the anti-osteogenic effect induced by persistent TNF-*α* supplementation. This observation is of paramount importance if we consider that defective bone repair processes, such as delayed new bone formation or excessive bone resorption, are often strictly associated to prolonged inflammatory states [64,65]. These preliminary in vitro results, therefore, may indicate that HBO could be successfully employed in all those conditions of inflammation-related bone loss. In line with this hypothesis, the recent work of Bosco and colleagues demonstrated that two cycles of HBO therapy on 23 patients with avascular necrosis of the femoral head resulted in a significant reduction of TNF-*α* and IL-6 plasma levels over time, thus suggesting that HBO may have an anti-inflammatory activity [66].

In this study, we additionally investigated the effects of pressure and oxygen signals on osteogenic-committed cells, with the aim to elucidate whether HBO could have a role in the course of the hADSCs osteogenic differentiation process. For this purpose, hADSCs were pre-differentiated for 10 days in ODM before starting the HBO, HB or HO treatments. This step produces cells that are committed to the osteogenic lineage but not yet undergoing middle or late stages of osteoblastic differentiation [67]. By comparing the data of this experimental scenario with those exposed so far, it is possible to clarify if the pressure and oxygen signals have a greater influence on the undifferentiated cells or on partially differentiated cells. Interestingly, at both 14 and 21 days of culture, HBO did not affect cell proliferation and matrix mineralization of hADSCs pre-exposed to osteogenic stimuli when compared to the untreated condition; this was also true for cells subjected to the HB or HO treatments alone. We can therefore hypothesize that undifferentiated and osteogenically pre-committed cells respond differently to pressure and oxygen signals, in particular under an inflammatory environment. Our data would support the hypothesis that HBO, and its individual constituents, only affect the behavior of cells uncommitted to the osteogenic lineage. On the contrary, high pressure and high oxygen do not seem to influence the biological properties of hADSCs if already pre-committed to the osteogenic phenotype. Remarkably, the increase in osteogenic differentiation observed in hADSCs treated with HBO in the presence of pro-inflammatory mediators, accompanied by a reduction in cell proliferation of these cells, would provide an indication of the potential application of HBO therapy in all those inflammatory processes requiring bone regeneration.

Besides phenotypic characteristics like extracellular matrix mineralization, the effect of the HBO treatment was investigated at molecular level, by measuring the expression of genes that control proper osteoblast differentiation and matrix mineralization. The gene expressions of several key markers were quantified over 21 days to determine the influence of oxygen and pressure signals at different stages of the hADSCs osteogenic differentiation process. The expression of the transcription factors RUNX2 and OSX has been shown to be necessary for osteoblast differentiation at a relatively early stage, with OSX acting downstream of RUNX2 [68,69]. RUNX2 is known to regulate the downstream expression of many others phenotypic markers, among which are ALP, OCN, and OPN [70,71]. In control cells committed to the osteogenic lineage, RUNX2 mRNA levels were stable from day 7 to day 21, whereas OSX expression was low in the early stage but increased with time during differentiation, consistently with published works [72,73]. In contrast, ALP expression reached the highest peak after 14 days of differentiation, indicative of the osteogenic maturation process, then it decreased at day 21. In agreement with previous studies, ALP gene expression has its maximum at around day 14 during osteogenic differentiation; then, as the mineralization progresses, its expression undergoes a down-regulation due to the development of an osteoid matrix around osteoblasts [67,74]. Starting from day 14, the osteogenic-differentiating cells also began to express OCN, in agreement with the findings that OCN appears concomitantly with the mineralization phase of bone formation [72]. Also the increment of OPN expression over time well correlates with data available in the literature [53]. When hADSCs were exposed to HBO for all the duration of differentiation, a certain reduction in the expression profile of some of the above described markers was evidenced. For example, RUNX2 expression levels progressively increased throughout the 21-day culture period, along with those of OCN and OPN. Nevertheless, these levels were greatly lower than control at all time points of the experiment. In addition, the peak in ALP expression was seen at day 21, thus suggesting that the combination of elevated pressure and elevated oxygen possibly determines a delay in the osteogenic differentiation process of hADSCs. By contrast, when hADSCs were HBO-treated after a 10-day osteogenic differentiation period, the analysis of gene expression on the same markers did not show any differential change (data not shown), which would confirm the MTT and ARS quantification data presented above.

Considering the stimuli individually, hyperoxia without pressure had a more similar effect to HBO on hADSCs osteogenesis than HB alone. In fact, the expression profile of most of the genes examined trended the same in the HBO- and HO-treated groups. Previous studies have shown that changes in oxygen tension rather than in pressure directly impact on bone cell functions [75,76,77]. When evaluating the expression profile of the osteogenic markers in cells exposed to in vitro simulated inflammatory conditions, less pronounced differences between control and treated groups were seen. These differences were mainly present at early stages of osteogenic differentiation, as in the case of RUNX2, OSX and ALP, but disappeared at later time points. During bone formation, osteogenesis is spatially and temporally coupled to the neovascularization process [78]. VEGF is involved in both processes, being the most specific growth factor for neovascularization, and a downstream target of OSX [79,80]. Its expression is low at the beginning of osteoblast differentiation, it increases in parallel with OCN expression during terminal differentiation, then it peaks during mineralization [81]. Our results are in agreement with previous studies demonstrating that VEGF synthesis increases following HBO treatment [79]. In particular, this increment was visible at the final stage of osteogenic differentiation when cells were grown in ODM (day 21), and at earlier time points (day 7) in the presence of inflammation. The increase in VEGFA expression was accompanied by up-regulation of KDR, one of its two receptors [82,83].

Overall, these observations would strengthen the hypothesis that HBO is able to counteract the anti-osteogenic effect induced by the cytokine supplementation. Looking at the literature, multiple signaling pathways, including Transforming Growth Factor-*β*, Fibroblast Growth Factor, Hedgehog, Ephrin, and sympathetic signaling pathways, are known to play important roles during osteogenic differentiation of MSCs [72]. Among these, the canonical Wnt pathway (Wnt3a/*β*-catenin) appears to be particularly important for bone biology, with RUNX2 being a direct target [84,85]. In the absence of a Wnt ligand, cytoplasmic *β*-catenin is phosphorylated by glycogen synthase kinase 3*β* (GSK3*β*), and this leads to its degradation via the ubiquitin/proteasome pathway [86]. Conversely, in the presence of Wnt proteins, GSK3*β* is inhibited resulting in unphosphorylated *β*-catenin, which is therefore not degraded and accumulates in the cytoplasm. Then, *β*-catenin enters the nucleus where it affects gene expression through binding to transcription factors of the TCF/LEF family. It has been demonstrated that the canonical Wnt pathway is modulated by oxygen availability in the cell microenvironment [87]. Also, the binding of TNF-α to its receptors results in the activation of multiple signaling pathways, which interact with each other to regulate the proliferation and differentiation of MSCs [62]. Interestingly, some of the pathways activated by TNF-*α* are implicated in the phosphorilation of GSK3*β*. It has been shown that GSK3*β* is required for TNF-*α*-mediated inhibition of osteogenic differentiation in MSCs [88]. In addition, TNF-*α* was found to suppress MSC osteogenesis through regulation of the Wnt signaling pathway at different levels, including inhibition of *β*-catenin nuclear accumulation, decreased *β*-catenin and reduced expression of Wnt proteins [58]. In the light of these considerations, further work could involve, for example, the treatment of hADSCs with siRNA targeting molecules of the Wnt/*β*-catenin pathway in the presence of high oxygen and/or TNF-*α*. In this way, it might be possible to unravel whether the two stimuli share common molecular mediators, and to what extent.

## 4. Materials and Methods

### 4.1. Ethic Statement

Adipose tissue was obtained during abdominoplasty procedures from the abdominal region of patients who had given written informed consent, according to the guidelines of the University of Padova’s Plastic Surgery Clinic. All experiments were approved by the ethical committee of the University of Padova (5 March 2009; 20150) and were conducted in accordance with the ethical standards of the Helsinki Declaration.

### 4.2. Isolation and Culture of hADSCs

hADSCs were isolated from adipose tissue samples as published elsewhere [89]. Briefly, the fat was washed with Phosphate Buffered Saline (PBS, EuroClone, Rome, Italy) and cut into small pieces. The tissue was then digested with a solution of 0.075% Collagenase from Clostridium histolyticum Type II (Sigma-Aldrich, Saint Louis, MA, USA) in Hank’s Balanced Salts Solution (HBSS, Lonza, Italy) and placed under stirring for 3 h at room temperature. Collagenase’s activity was neutralized with an equal volume of complete Dulbecco’s Modified Eagle Medium made by DMEM High Glucose (EuroClone), 10% Fetal Bovine Serum (FBS, EuroClone) and 1% Penicillin/Streptomycin (P/S, EuroClone), and centrifuged at 260 × *g* for 4 min. The cell pellet was seeded in 75 cm^2^ cell culture flasks (Corning Incorporated, Corning, New York, NY, USA) at a density of 5 × 10^3^ cells/cm^2^ with cDMEM and cultured at 37 °C and 5% CO_2_. At 80–90% confluence, cells were detached with trypsin-EDTA solution (Sigma-Aldrich) and passaged repeatedly. hADSCs between passages 3 and 5 were used in this study.

### 4.3. Experimental Designs

**Figure 8:**
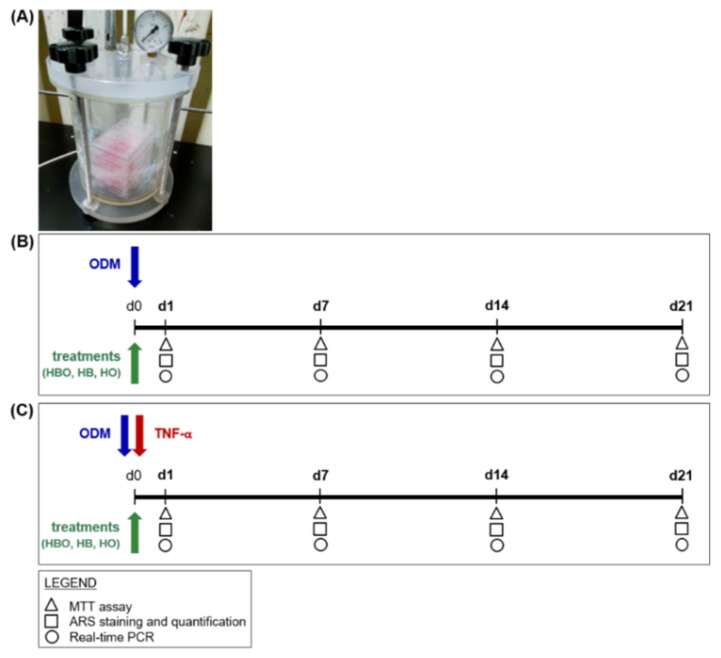
Schematic experimental design of treatments starting simultaneously with the osteogenic differentiation of hADSCs. (**A**) Image of hyperbaric culture chamber used for HBO exposures. (**B**) hADSCs are grown in ODM and contextually subjected to one of the following treatments: HBO, HB, or HO. The control condition is represented by cells grown under normoxia and normobarism. Each treatment is performed daily for a total of 21 days. (**C**) hADSCs are treated as described above but in the presence of 10 ng/mL TNF-*α*. Tests for evaluating cell metabolic activity and osteogenic differentiation, as evidenced in the legend, are performed at d1, d7, d14, and d21.

An overview of the first experimental design is depicted in Figure 8. hADSCs were seeded in 24 Well Clear TC-Treated Multiple Well Plates (Corning Incorporated) at a density of 2 × 10^4^ cells/cm^2^. Cells were then cultured in the presence of ODM, which consisted of cDMEM supplemented with 10 nM dexamethasone (Sigma-Aldrich), 10 ng/mL Fibroblast Growth Factor 2 (ProSpec, East Brunswick, NJ, USA), and 10 mM beta-glicerophosphate (Sigma-Aldrich). Contextually to the beginning of differentiation, the cells were exposed daily for 60 min, and up to 21 days, to one of the following treatments into appropriate designed chambers (Costruzioni Riunite Moro, CRM, Treviso, Italy) prepared at the Hyperbaric Medicine Center (Padova, Italy) (Figure 8A): hyperbarism (2.4 ATA) and hyperoxia (100% O_2_), in the text referred to as HBO; hyperbarism (2.4 ATA) and normoxia (21% O_2_), indicated as HB; normobarism (0.97 ATA) and hyperoxia (100% O_2_), called HO (Figure 8B). The chambers were flushed twice with ambient air at up to 2 ATA for 10 min, before starting the different treatment sessions. Cell incubations were performed at 37 °C and 5% CO_2_, with medium changes every 3 days. In order to simulate a pro-inflammatory environment in vitro, ODM was supplemented with 10 ng/mL of TNF-*α* (ProSpec); then, the cells were subjected to the same different treatments as listed above (Figure 8C). TNF-*α* was freshly added in the medium at each change. Untreated cells cultured in a standard humidified incubator at 37 °C containing 5% CO_2_, in the presence of ODM with or without inflammation, represented the control condition (CTRL).

**Figure 9:**
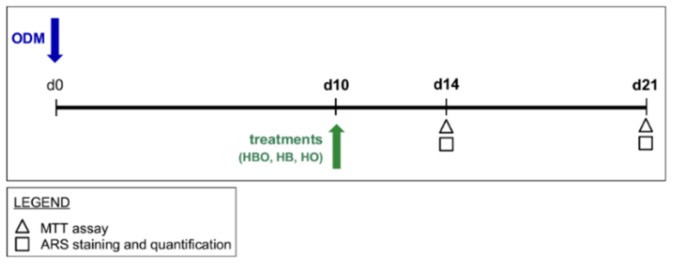
Schematic experimental design of treatments starting after osteogenic pre-commitment of hADSCs. hADSCs are pre-differentiated in ODM for 10 days (d10) before treatment with HBO, HB, or HO. Each treatment is performed daily for 60 min from the tenth day to d21 of culture. Tests for evaluating cell metabolic activity and osteogenic differentiation, as evidenced in the legend, are performed at d14 and d21.

In the second experimental design, hADSCs (2 × 10^4^ cells/cm^2^) were pre-stimulated with osteogenic factors for 10 days. Then, treatments with HBO, HB, or HO started from the tenth day to d21 of culture (Figure 9).

### 4.4. MTT Assay

To determine the cell metabolic activity under the different experimental conditions, the MTT-based viability assay was performed, as already published [90]. Briefly, after removing the culture medium and washing with PBS, cells were incubated with 1 mL of 0.5 mg/mL MTT (Sigma-Aldrich) solution prepared in PBS for 3 h at 37 °C. After removal of the MTT solution, 0.5 mL of 10% dimethyl sulfoxide in isopropanol was added to extract the formazan in the samples for 10 min at 37 °C. For each sample, Optical Density (O.D.) values at 570 nm were recorded in duplicate on 200 μL aliquots using a multilabel plate reader (Victor 3, Perkin Elmer, Milan, Italy).

### 4.5. DNA Content

Total DNA was isolated from cells after 1 day, 7 days, 14 days and 21 days of culture using the DNeasy Blood and Tissue kit (Qiagen, Hilden, Germany), following the manufacturer’s protocol for tissue isolation, as published elsewhere [91]. The DNA concentration was measured with NanoDrop™ ND-1000 (Thermo Fisher Scientific, Waltham, MA, USA). Cell number was then extrapolated from a standard curve, previously generated by relating the number of cells (increasing quantity) to the corresponding DNA content.

### 4.6. ARS Staining and Quantification

ARS staining was used to reveal the ability of hADSCs to deposit a mineral matrix that is a characteristic of osteoblastic lineage. Firstly, the cells were fixed with 10% formalin for 10 min at room temperature. Cells were then washed twice with PBS, air dried, and stored at −20 °C till further use. For ARS staining, cells were washed twice with double-distilled water (ddH_2_O) and incubated with 0.5 mL of 40 mM ARS solution (pH 4.2) for 20 min at room temperature with gentle shaking. After removing the solution, cells were washed with ddH_2_O 4 times. Then cells were washed with PBS and incubated with 0.5 mL of 10 mM CPC (Sigma-Aldrich) in 10 mM sodium phosphate solution (pH 7,0) for 20 min at room temperature with gentle agitation. 200 uL of the solution was transferred to 96-wells plate and measured at 570 nm in a plate reader.

### 4.7. Real-Time PCR

Total RNA was isolated after 7, 14, and 21 days of culture with the total RNA purification Plus kit (Norgen Biotek, Thorold, ON, Canada). The RNA quality and concentration of the samples were measured using the NanoDrop™ ND-1000 (Thermo Fisher Scientific). The cDNA was synthesized following the SensiFAST™ cDNA Synthesis Kit (Bioline, Singapore). For each sample, 1000 ng of total RNA was retrotranscribed in a reaction volume of 20 µL. Real-time PCR was then performed on a Rotor-Gene 3000 (Corbett Research, Sydney, Australia). A total of 1.5 µL cDNA at the appropriate dilution was added to the final reaction volume of 15 µL. The reaction contained 1× SensiFAST™ SYBR^®^No-ROX mix (Bioline), forward and reverse primer (400 nM each), and water. Human primers were selected for each target gene with Primer 3 software (Table 1). The following PCR program was used: 95 °C for 2 min, followed by 45 cycles of 95 °C for 5 s, 60 °C for 10 s and 72 °C for 20 s. Differences in gene expression were calculated by normalizing to the expression of the transferrin receptor (TFRC) housekeeping gene.

### 4.8. Statistical Analysis

Each experiment was performed independently three times in triplicate. Statistical calculations were performed using GraphPad software (La Jolla, CA, USA). The mean values for quantitative data were compared applying non-parametric Kruskal-Wallis test for MTT and ARS assays results as well as for DNA content measurements. Real-time PCR data were analyzed using Student’s unpaired t-test based on 2deltaCt values for each gene of the test group compared with that of the control group. Significance levels were calculated in comparison to the control condition and indicated as follows: *p* < 0.05, *p* < 0.01, *p* < 0.001.

## 5. Conclusions

In conclusion, the results of our work demonstrate that hADSCs respond differently to pressure and oxygen signals in the course of the osteogenic differentiation process. HBO, and its individual constituents, elevated pressure and elevated oxygen, do not affect the metabolic activity and the osteogenic properties of hADSCs if cells are pre-committed to the osteogenic lineage. On the contrary, when the HBO treatment starts contextually to the osteogenic differentiation of the cells, a decrease in cell proliferation is observed in the middle stages of culture, together with a reduction in extracellular calcium deposition and expression of osteogenic markers at later time points. High oxygen alone shows effects comparable to HBO, although less pronounced, whereas high pressure alone has no effect, similarly to untreated control cells. Our results also reveal that constant exposure to the pro-inflammatory cytokine TNF-*α* clearly inhibits hADSCs osteogenesis. Nevertheless, if prolonged inflammation is combined with high pressure and high oxygen stimuli, enhanced mineral deposition and expression of osteogenic markers by hADSCs are displayed at later time points. This effect is not observed in cells exposed to HB or HO alone. Considering that prolonged inflammatory states, which are typical of several bone disorders, have destructive effects on bone tissue formation, resolution of inflammation through HBO therapy might be beneficial for hADSCs osteogenesis. Although further studies are necessary, the findings of the present work would suggest that HBO could have a promising future as an adjunctive therapeutic approach for treating many challenges cases of inflammation-related bone loss.

## Figures and Tables

**Table 1:** Human primer sequences.

Gene Name	Forward Primer (5′ → 3′)	Reverse Primer (5′ → 3′)	Amplicon Size (bp)
ALP ^1^	GGCTTCTTCTTGCTGGTGGA	CAAATGTGAAGACGTGGGAATGG	181
KDR ^2^	GGAGGAGGAGGAAGTATGTGACC	AACCATACCACTGTCCGTCTG	184
OCN ^3^	GCAGCGAGGTAGTGAAGAGAC	AGCAGAGCGACACCCTA	193
OPN ^4^	TGGAAAGCGAGGAGTTGAATGG	GCTCATTGCTCTCATCATTGGC	192
OSX ^5^	TCAGAATCTCAGTTGATAGGGTTTCTC	GGGTACATTCCAGTCCTTCTCC	183
RANKL ^6^	TCAGCATCGAGGTCTCCAAC	CCATGCCTCTTAGTAGTCTCACA	194
RUNX2 ^7^	AGCCTTACCAAACAACACAACAG	CCATATGTCCTCTCAGCTCAGC	175
TFRC ^8^	TGTTTGTCATAGGGCAGTTGGAA	ACACCCGAACCAGGAATCTC	222
VEGFA ^9^	GGACAGAAAGACAGATCACAGGTAC	GCAGGTGAGAGTAAGCGAAGG	182

^1^ alkaline phosphatase, liver/bone/kidney; ^2^ kinase insert domain receptor; ^3^ osteocalcin; 4 osteopontin; ^5^ osterix; ^6^ tumor necrosis factor (ligand) superfamily, member 11; ^7^ runt related transcription factor 2; ^8^ transferrin receptor; ^9^ vascular endothelia growth factor A.

## References

[1] Loi F., Córdova L.A., Pajarinen J., Lin T.H., Yao Z., Goodman S.B. (2016). Inflammation, fracture and bone repair. Bone.

[2] Pirosa A., Gottardi R., Alexander P.G., Tuan R.S. (2018). Engineering in-vitro stem cell-based vascularized bone models for drug screening and predictive toxicology. Stem Cell Res. Ther..

[3] Bennett M.H., Stanford R., Turner R. (2005). Hyperbaric oxygen therapy for promoting fracture healing and treating fracture non-union. Cochrane Database Syst. Rev..

[4] Fang R.C., Galiano R.D. (2009). Adjunctive therapies in the treatment of osteomyelitis. Semin. Plast. Surg..

[5] Devaraj D., Srisakthi D. (2014). Hyperbaric oxygen therapy—Can it be the new era in dentistry?. J. Clin. Diagn. Res..

[6] Vezzani G., Quartesan S., Cancellara P., Camporesi E., Mangar D., Bernasek T., Dalvi P., Yang Z., Paoli A., Rizzato A. (2017). Hyperbaric oxygen therapy modulates serum OPG/RANKL in femoral head necrosis patients. J. Enzyme Inhib. Med. Chem..

[7] Bennett M., Best T.M., Babul S., Taunton J., Lepawsky M. (2005). Hyperbaric oxygen therapy for delayed onset muscle soreness and closed soft tissue injury. Cochrane Database Syst. Rev..

[8] Gurdol F., Cimsit M., Oner-Iyidogan Y., Kocak H., Sengun S., Yalcinkaya-Demirsoz S. (2010). Collagen synthesis, nitric oxide and asymmetric dimethylarginine in diabetic subjects undergoing hyperbaric oxygen therapy. Physiol. Res..

[9] Zhang Q., Chang Q., Cox R.A., Gong X., Gould L.J. (2008). Hyperbaric oxygen attenuates apoptosis and decreases inflammation in an ischemic wound model. J. Invest. Dermatol..

[10] Marsell R., Einhorn T.A. (2011). The biology of fracture healing. Injury.

[11] Liu H., Li D., Zhang Y., Li M. (2018). Inflammation, mesenchymal stem cells and bone regeneration. Histochem. Cell Biol..

[12] Bernardo M.E., Fibbe W.E. (2013). Mesenchymal stromal cells: Sensors and switchers of inflammation. Cell Stem Cell.

[13] Mountziaris P.M., Mikos A.G. (2008). Modulation of the inflammatory response for enhanced bone tissue regeneration. Tissue Eng. Part. B. Rev..

[14] Zhang Q., Chen B., Zhu D., Yan F. (2016). Biomarker levels in gingival crevicular fluid of subjects with different periodontal conditions: A cross-sectional study. Arch. Oral Biol..

[15] Friedewald V.E., Kornman K.S., Beck J.D., Genco R., Goldfine A., Libby P., Offenbacher S., Ridker P.M., Van Dyke T.E., Roberts W.C. (2009). The American Journal of Cardiology and Journal of Periodontology editors’ consensus: Periodontitis and atherosclerotic cardiovascular disease. J. Periodontol..

[16] Isola G., Polizzi A., Santonocito S., Alibrandi A., Ferlito S. (2019). Expression of Salivary and Serum Malondialdehyde and Lipid Profile of Patients with Periodontitis and Coronary Heart Disease. Int. J. Mol. Sci..

[17] Isola G., Polizzi A., Alibrandi A., Indelicato F., Ferlito S. (2020). Analysis of Endothelin-1 Concentrations in Individuals with Periodontitis. Sci. Rep..

[18] Isola G., Polizzi A., Muraglie S., Leonardi R., Lo Giudice A. (2019). Assessment of Vitamin C and Antioxidant Profiles in Saliva and Serum in Patients with Periodontitis and Ischemic Heart Disease. Nutrients.

[19] Isola G., Alibrandi A., Currò M., Matarese M., Ricca S., Matarese G., Ientile R., Kocher T. (2020). Evaluation of salivary and serum ADMA levels in patients with periodontal and cardiovascular disease as subclinical marker of cardiovascular risk. J. Periodontol..

[20] Vindigni V., Giatsidis G., Reho F., Dalla Venezia E., Mammana M., Bassetto F., Andrades J.A. (2013). Adipose Derived Stem Cells: Current State of the Art and Prospective Role in Regenerative Medicine and Tissue Engineering. Regenerative Medicine and Tissue Engineering.

[21] Simonacci F., Bertozzi N., Raposio E. (2017). Off-label use of adipose-derived stem cells. Ann. Med. Surg..

[22] Zanetti A.S., Sabliov C., Gimble J.M., Hayes D.J. (2013). Human adipose-derived stem cells and three-dimensional scaffold constructs: A review of the biomaterials and models currently used for bone regeneration. J. Biomed. Mater. Res. B. Appl. Biomater..

[23] Gardin C., Ferroni L., Bellin G., Rubini G., Barosio S., Zavan B. (2018). Therapeutic Potential of Autologous Adipose-Derived Stem Cells for the Treatment of Liver Disease. Int. J. Mol. Sci..

[24] Ferroni L., Gardin C., Bellin G., Vindigni V., Pavan C., Zavan B. (2019). Effects of novel antidepressant drugs on mesenchymal stem cell physiology. Biomed. Pharmacother..

[25] Wu D., Malda J., Crawford R., Xiao Y. (2007). Effects of hyperbaric oxygen on proliferation and differentiation of osteoblasts from human alveolar bone. Connect. Tissue Res..

[26] Al Hadi H., Smerdon G.R., Fox S.W. (2015). Hyperbaric oxygen therapy accelerates osteoblast differentiation and promotes bone formation. J. Dent..

[27] Lin S.S., Ueng S.W., Niu C.C., Yuan L.J., Yang C.Y., Chen W.J., Lee M.S., Chen J.K. (2014). Hyperbaric oxygen promotes osteogenic differentiation of bone marrow stromal cells by regulating Wnt3a/*β*-catenin signaling: An in vitro and in vivo study. Stem Cell Res..

[28] Lin S.S., Ueng S.W., Niu C.C., Yuan L.J., Yang C.Y., Chen W.J., Lee M.S., Chen J.K. (2014). Effects of hyperbaric oxygen on the osteogenic differentiation of mesenchymal stem cells. BMC Musculoskelet Disord.

[29] Hsieh C.P., Chiou Y.L., Lin C.Y. (2010). Hyperbaric oxygen-stimulated proliferation and growth of osteoblasts may be mediated through the FGF-2/MEK/ERK 1/2/NF-κB and PKC/JNK pathways. Connect. Tissue Res..

[30] Ferroni L., Gardin C., Dolkart O., Salai M., Barak S., Piattelli A., Amir-Barak H., Zavan B. (2018). Pulsed electromagnetic fields increase osteogenetic commitment of MSCs via the mTOR pathway in TNF-α mediated inflammatory conditions: An in-vitro study. Sci. Rep..

[31] Mohyeldin A., Garzón-Muvdi T., Quiñones-Hinojosa A. (2010). Oxygen in stem cell biology: A critical component of the stem cell niche. Cell Stem Cell.

[32] Barata P., Cervaens M., Resende R., Camacho O., Marques F. (2011). Hyperbaric oxygen effects on sports injuries. Ther. Adv. Musculoskelet Dis..

[33] Brahimi-Horn M.C., Pouysségur J. (2007). Oxygen, a source of life and stress. FEBS Lett..

[34] Fotia C., Massa A., Boriani F., Baldini N., Granchi D. (2015). Hypoxia enhances proliferation and stemness of human adipose-derived mesenchymal stem cells. Cytotechnology.

[35] Buravkova L.B., Grinakovskaia O.S., Andreeva E.P., Zhambalova A.P., Kozionova M.P. (2009). Characteristics of human lipoaspirate-isolated mesenchymal stromal cells cultivated under a lower oxygen tension. Tsitologiia.

[36] Basciano L., Nemos C., Foliguet B., de Isla N., de Carvalho M., Tran N., Dalloul A. (2011). Long term culture of mesenchymal stem cells in hypoxia promotes a genetic program maintaining their undifferentiated and multipotent status. BMC Cell Biol..

[37] Malladi P., Xu Y., Chiou M., Giaccia A.J., Longaker M.T. (2006). Effect of reduced oxygen tension on chondrogenesis and osteogenesis in adipose-derived mesenchymal cells. Am. J. Physiol. Cell Physiol..

[38] Fehrer C., Brunauer R., Laschober G., Unterluggauer H., Reitinger S., Kloss F., Gülly C., Gassner R., Lepperdinger G. (2007). Reduced oxygen tension attenuates differentiation capacity of human mesenchymal stem cells and prolongs their lifespan. Aging Cell.

[39] Pattappa G., Thorpe S.D., Jegard N.C., Heywood H.K., de Bruijn J.D., Lee D.A. (2013). Continuous and uninterrupted oxygen tension influences the colony formation and oxidative metabolism of human mesenchymal stem cells. Tissue Eng. Part. C Methods.

[40] Körpınar Ş., Uzun H. (2019). The Effects of Hyperbaric Oxygen at Different Pressures on Oxidative Stress and Antioxidant Status in Rats. Medicina (Kaunas).

[41] Liska G.M., Lippert T., Russo E., Nieves N., Borlongan C.V. (2018). A Dual Role for Hyperbaric Oxygen in Stroke Neuroprotection: Preconditioning of the Brain and Stem Cells. Cond Med..

[42] Fosen K.M., Thom S.R. (2014). Hyperbaric oxygen, vasculogenic stem cells, and wound healing. Antioxid Redox Signal..

[43] Dennog C., Gedik C., Wood S., Speit G. (1999). Analysis of oxidative DNA damage and HPRT mutations in humans after hyperbaric oxygen treatment. Mutat. Res..

[44] Thom S.R. (2009). Oxidative stress is fundamental to hyperbaric oxygen therapy. J. Appl. Physiol. (1985).

[45] Gao Z.X., Rao J., Li Y.H. (2017). Hyperbaric oxygen preconditioning improves postoperative cognitive dysfunction by reducing oxidant stress and inflammation. Neural. Regen. Res..

[46] Denizot F., Lang R. (1986). Rapid colorimetric assay for cell growth and survival. Modifications to the tetrazolium dye procedure giving improved sensitivity and reliability. J. Immunol. Methods.

[47] Wong A.K., Schönmeyr B.H., Soares M.A., Li S., Mehrara B.J. (2008). Hyperbaric oxygen inhibits growth but not differentiation of normal and irradiated osteoblasts. J. Craniofac. Surg..

[48] Bastidas-Coral A.P., Bakker A.D., Zandieh-Doulabi B., Kleverlaan C.J., Bravenboer N., Forouzanfar T., Klein-Nulend J. (2016). Cytokines TNF-α, IL-6, IL-17F, and IL-4 Differentially Affect Osteogenic Differentiation of Human Adipose Stem Cells. Stem Cells Int..

[49] Egea V., von Baumgarten L., Schichor C., Berninger B., Popp T., Neth P., Goldbrunner R., Kienast Y., Winkler F., Jochum M. (2011). TNF-α respecifies human mesenchymal stem cells to a neural fate and promotes migration toward experimental glioma. Cell Death Differ..

[50] Benincasa J.C., de Freitas Filho L.H., Carneiro G.D., Sielski M.S., Giorgio S., Werneck C.C., Vicente C.P. (2019). Hyperbaric oxygen affects endothelial progenitor cells proliferation in vitro. Cell Biol. Int..

[51] Wang X., Liu J., Jiang L., Wei X., Niu C., Wang R., Zhang J., Meng D., Yao K. (2016). Bach1 Induces Endothelial Cell Apoptosis and Cell-Cycle Arrest through ROS Generation. Oxid. Med. Cell Longev..

[52] Chandel N.S., Budinger G.R. (2007). The cellular basis for diverse responses to oxygen. Free Radic Biol. Med..

[53] Matta C., Szűcs-Somogyi C., Kon E., Robinson D., Neufeld T., Altschuler N., Berta A., Hangody L., Veréb Z., Zákány R. (2019). Osteogenic differentiation of human bone marrow-derived mesenchymal stem cells is enhanced by an aragonite scaffold. Differentiation.

[54] Croes M., Oner F.C., Kruyt M.C., Blokhuis T.J., Bastian O., Dhert W.J., Alblas J. (2015). Proinflammatory Mediators Enhance the Osteogenesis of Human Mesenchymal Stem Cells after Lineage Commitment. PLoS ONE.

[55] Kim S., Shin M.Y., Son K.H., Sohn H.Y., Lim J.H., Lee J.H., Kwun I.S. (2014). Yam (*Dioscorea batatas*) Root and Bark Extracts Stimulate Osteoblast Mineralization by Increasing Ca and P Accumulation and Alkaline Phosphatase Activity. Prev. Nutr. Food Sci..

[56] Hess K., Ushmorov A., Fiedler J., Brenner R.E., Wirth T. (2009). TNFalpha promotes osteogenic differentiation of human mesenchymal stem cells by triggering the NF-kappaB signaling pathway. Bone.

[57] Lu Z., Wang G., Dunstan C.R., Chen Y., Lu W.Y., Davies B., Zreiqat H. (2013). Activation and promotion of adipose stem cells by tumour necrosis factor-α preconditioning for bone regeneration. J. Cell Physiol..

[58] Gilbert L.C., Chen H., Lu X., Nanes M.S. (2013). Chronic low dose tumor necrosis factor-α (TNF) suppresses early bone accrual in young mice by inhibiting osteoblasts without affecting osteoclasts. Bone.

[59] Abuna R.P., De Oliveira F.S., Santos T.e.S., Guerra T.R., Rosa A.L., Beloti M.M. (2016). Participation of TNF-α in Inhibitory Effects of Adipocytes on Osteoblast Differentiation. J. Cell Physiol..

[60] Lacey D.C., Simmons P.J., Graves S.E., Hamilton J.A. (2009). Proinflammatory cytokines inhibit osteogenic differentiation from stem cells: Implications for bone repair during inflammation. Osteoarthr. Cartil..

[61] Ding J., Ghali O., Lencel P., Broux O., Chauveau C., Devedjian J.C., Hardouin P., Magne D. (2009). TNF-alpha and IL-1beta inhibit RUNX2 and collagen expression but increase alkaline phosphatase activity and mineralization in human mesenchymal stem cells. Life Sci..

[62] Huang H., Zhao N., Xu X., Xu Y., Li S., Zhang J., Yang P. (2011). Dose-specific effects of tumor necrosis factor alpha on osteogenic differentiation of mesenchymal stem cells. Cell Prolif..

[63] Qin Z., Fang Z., Zhao L., Chen J., Li Y., Liu G. (2015). High dose of TNF-α suppressed osteogenic differentiation of human dental pulp stem cells by activating the Wnt/β-catenin signaling. J. Mol. Histol..

[64] Wu X., Xu W., Feng X., He Y., Liu X., Gao Y., Yang S., Shao Z., Yang C., Ye Z. (2015). TNF-a mediated inflammatory macrophage polarization contributes to the pathogenesis of steroid-induced osteonecrosis in mice. Int. J. Immunopathol. Pharmacol..

[65] Lange U., Dischereit G., Neumann E., Frommer K., Tarner I.H., Ulf M.-L.K.-K. (2015). Osteoimmunological Aspects on Inflammation and Bone Metabolism. J. Rheum. Dis. Treat..

[66] Bosco G., Vezzani G., Mrakic Sposta S., Rizzato A., Enten G., Abou-Samra A., Malacrida S., Quartesan S., Vezzoli A., Camporesi E. (2018). Hyperbaric oxygen therapy ameliorates osteonecrosis in patients by modulating inflammation and oxidative stress. J. Enzyme Inhib. Med. Chem..

[67] Eijken M., Koedam M., van Driel M., Buurman C.J., Pols H.A., van Leeuwen J.P. (2006). The essential role of glucocorticoids for proper human osteoblast differentiation and matrix mineralization. Mol. Cell Endocrinol..

[68] Ducy P., Zhang R., Geoffroy V., Ridall A.L., Karsenty G. (1997). Osf2/Cbfa1: A transcriptional activator of osteoblast differentiation. Cell.

[69] Nakashima K., Zhou X., Kunkel G., Zhang Z., Deng J.M., Behringer R.R., de Crombrugghe B. (2002). The novel zinc finger-containing transcription factor osterix is required for osteoblast differentiation and bone formation. Cell.

[70] Anderson J.M., Vines J.B., Patterson J.L., Chen H., Javed A., Jun H.W. (2011). Osteogenic differentiation of human mesenchymal stem cells synergistically enhanced by biomimetic peptide amphiphiles combined with conditioned medium. Acta Biomater..

[71] Liu T.M., Lee E.H. (2013). Transcriptional regulatory cascades in Runx2-dependent bone development. Tissue Eng. Part. B Rev..

[72] Huang W., Yang S., Shao J., Li Y.P. (2007). Signaling and transcriptional regulation in osteoblast commitment and differentiation. Front. Biosci..

[73] Chen Y.Q., Liu Y.S., Liu Y.A., Wu Y.C., Del Álamo J.C., Chiou A., Lee O.K. (2016). Bio- chemical and physical characterizations of mesenchymal stromal cells along the time course of directed differentiation. Sci. Rep..

[74] Chen J., Deng L., Porter C., Alexander G., Patel D., Vines J., Zhang X., Chasteen-Boyd D., Sung H.J., Li Y.P. (2018). Angiogenic and Osteogenic Synergy of Human Mesenchymal Stem Cells and Human Umbilical Vein Endothelial Cells Cocultured on a Nanomatrix. Sci. Rep..

[75] Tuncay O.C., Ho D., Barker M.K. (1994). Oxygen tension regulates osteoblast function. Am. J. Orthod. Dentofacial. Orthop..

[76] Lu C., Saless N., Wang X., Sinha A., Decker S., Kazakia G., Hou H., Williams B., Swartz H.M., Hunt T.K. (2013). The role of oxygen during fracture healing. Bone.

[77] Al Hadi H., Smerdon G.R., Fox S.W. (2013). Hyperbaric oxygen therapy suppresses osteoclast formation and bone resorption. J. Orthop. Res..

[78] Wan C., Shao J., Gilbert S.R., Riddle R.C., Long F., Johnson R.S., Schipani E., Clemens T.L. (2010). Role of HIF-1alpha in skeletal development. Ann. N. Y. Acad. Sci..

[79] Sheikh A.Y., Gibson J.J., Rollins M.D., Hopf H.W., Hussain Z., Hunt T.K. (2000). Effect of hyperoxia on vascular endothelial growth factor levels in a wound model. Arch. Surg..

[80] Tang W., Yang F., Li Y., de Crombrugghe B., Jiao H., Xiao G., Zhang C. (2012). Transcriptional regulation of Vascular Endothelial Growth Factor (VEGF) by osteoblast-specific transcription factor Osterix (Osx) in osteoblasts. J. Biol. Chem..

[81] Deckers M.M., Karperien M., van der Bent C., Yamashita T., Papapoulos S.E., Löwik C.W. (2000). Expression of vascular endothelial growth factors and their receptors during osteoblast differentiation. Endocrinology.

[82] Patterson C., Perrella M.A., Hsieh C.M., Yoshizumi M., Lee M.E., Haber E. (1995). Cloning and functional analysis of the promoter for KDR/flk-1, a receptor for vascular endothelial growth factor. J. Biol. Chem..

[83] Yang Y., Wei H., Zhou X., Zhang F., Wang C. (2017). Hyperbaric oxygen promotes neural stem cell proliferation by activating vascular endothelial growth factor/extracellular signal-regulated kinase signaling after traumatic brain injury. Neuroreport.

[84] Westendorf J.J., Kahler R.A., Schroeder T.M. (2004). Wnt signaling in osteoblasts and bone diseases. Gene.

[85] Gaur T., Lengner C.J., Hovhannisyan H., Bhat R.A., Bodine P.V., Komm B.S., Javed A., van Wijnen A.J., Stein J.L., Stein G.S. (2005). Canonical WNT signaling promotes osteogenesis by directly stimulating Runx2 gene expression. J. Biol. Chem..

[86] Kühl S.J., Kühl M. (2013). On the role of Wnt/β-catenin signaling in stem cells. Biochim. Biophys. Acta.

[87] Mazumdar J., O’Brien W.T., Johnson R.S., LaManna J.C., Chavez J.C., Klein P.S., Simon M.C. (2010). O_2_ regulates stem cells through Wnt/β-catenin signalling. Nat. Cell Biol..

[88] Kong X., Liu Y., Ye R., Zhu B., Zhu Y., Liu X., Hu C., Luo H., Zhang Y., Ding Y. (2013). GSK3β is a checkpoint for TNF-α-mediated impaired osteogenic differentiation of mesenchymal stem cells in inflammatory microenvironments. Biochim. Biophys. Acta.

[89] Cecchinato F., Karlsson J., Ferroni L., Gardin C., Galli S., Wennerberg A., Zavan B., Andersson M., Jimbo R. (2015). Osteogenic potential of human adipose-derived stromal cells on 3-dimensional mesoporous TiO_2_ coating with magnesium impregnation. Mater. Sci Eng. C Mater. Biol. Appl..

[90] Gardin C., Ricci S., Ferroni L., Guazzo R., Sbricoli L., De Benedictis G., Finotti L., Isola M., Bressan E., Zavan B. (2015). Decellularization and Delipidation Protocols of Bovine Bone and Pericardium for Bone Grafting and Guided Bone Regeneration Procedures. PLoS ONE.

[91] Ghensi P., Bressan E., Gardin C., Ferroni L., Soldini M.C., Mandelli F., Soldini C., Zavan B. (2017). The Biological Properties of OGI Surfaces Positively Act on Osteogenic and Angiogenic Commitment of Mesenchymal Stem Cells. Materials (Basel).

